# Molecular evolution and expression divergence of the *Populus euphratica Hsf* genes provide insight into the stress acclimation of desert poplar

**DOI:** 10.1038/srep30050

**Published:** 2016-07-18

**Authors:** Jin Zhang, Huixia Jia, Jianbo Li, Yu Li, Mengzhu Lu, Jianjun Hu

**Affiliations:** 1 State Key Laboratory of Tree Genetics and Breeding, Key Laboratory of Tree Breeding and Cultivation of the State Forestry Administration, Research Institute of Forestry, Chinese Academy of Forestry, Beijing 100091, China; 2 Co-Innovation Center for Sustainable Forestry in Southern China, Nanjing Forestry University, Nanjing 210037, China

## Abstract

Heat shock transcription factor (Hsf) family is one of the most important regulators in the plant kingdom. Hsf has been demonstrated to be involved in various processes associated with plant growth, development as well as in response to hormone and abiotic stresses. In this study, we carried out a comprehensive analysis of *Hsf* family in desert poplar, *Populus euphratica*. Total of 32 genes encoding Hsf were identified and they were classified into three main classes (A, B, and C). Gene structure and conserved motif analyses indicated that the members in each class were relatively conserved. Total of 10 paralogous pairs were identified in *PeuHsf* family, in which nine pairs were generated by whole genome duplication events. *K*a/*K*s analysis showed that *PeuHsfs* underwent purifying selection pressure. In addition, various *cis*-acting elements involved in hormone and stress responses located in the promoter regions of *PeuHsfs*. Gene expression analysis indicated that several *PeuHsfs* were tissue-specific expression. Compared to *Arabidopsis*, more *PeuHsf* genes were significantly induced by heat, drought, and salt stresses (21, 19, and 22 *PeuHsfs*, respectively). Our findings are helpful in understanding the distinguished adaptability of *P. euphratica* to extreme environment and providing a basis for functional analysis of *PeuHsfs* in the future.

As the terminal components of stress signal transduction chain, heat shock transcription factors (Hsfs) play an important role in mediating the expression of genes responsive to various abiotic stresses, especially heat stress (HS)^1^. Hsfs specifically recognize the binding motifs ‘AGAAnnTTCT’, called heat stress elements (HSEs) conserved in promoters of HS-inducible genes^2^.

The structure of Hsfs was similar to other transcription factors, a classical Hsf was composed by N-terminal highly conserved DNA-binding domain (DBD), oligomerization domain (OD), nuclear localization signal (NLS), nuclear export signal (NES), repressor domain (RD), and C-terminal activator peptide motif (AHA)^2^. The DBD is characterized by a central helix-turn-helix motif that specifically binds to the HSEs in promoters of target genes, this domain is the best preserved domain during the evolution^3^. The OD with a pattern of hydrophobic heptad repeats, referred to as the HR-A/B region, form a coiled-coil structure mediate oligomerization of Hsfs^2^. According to the length of the linker between DBD and HR-A/B regions and the amino acid length inserted into the HR-A/B region, plant Hsfs are grouped into three main classes (A, B, and C)^4^. The NLS and NES mediate the intracellular distribution, while activity of Hsfs depends on the shutting balance between nuclei and cytoplasm^5^. The RD are characterized by the tetrapeptide LFGV in the C-terminal of class B Hsfs, except HsfB5, which is function as repressor motif through interaction with the corepressor^6^. The C-terminal AHA motifs confer the transcriptional activator function of Hsfs and are class A Hsf specific and not found in class B or C^2^,^4^.

Numerous studies have indicated that Hsfs play important roles in plant responses to various environmental stresses, including heat, cold, drought, salinity, and oxidative stresses^3^. Within class A Hsf, the members of *HsfA1* subclass are considered as master regulators of HS response. The tomato *HsfA1a* has been reported as the single master regulator in thermotolerance^7^. And *HsfA1a* is required for the nuclear retention and transcriptional activation of *HsfA2*^8^. However, the situation might be inconsistent in different plant species. For example, single or multiple mutants of four *HsfA1s (a, b, d*, and *e*) revealed no such master *HsfA1* regulating the thermotolerance in *Arabidopsis*^9^. In many plant species, such as tomato, *Arabidopsis*, rice, *Populus trichocarpa*, and *Salix suchowensis, HsfA2* is the most strongly induced *Hsf* under heat stress^10^,^11^,^12^,^13^. In heat stressed cells, *HsfA2* becomes the dominant *Hsf* after long-term HS or repeated cycles of HS and recovery^14^. Although *HsfA2* can function to improve thermotolerance in the absence of *HsfA1s* when overexpressed in a quadruple knockout mutant *Arabidopsis (athsfA1a,b,d,e*)^15^. The hetero-oligomerization of HsfA1 and HsfA2 leads to form a superactivator complex, which activates downstream HS-inducible genes in a stronger manner than individuals^10^. In contrast, the HsfA4 and HsfA5 can also form oligomers, but their activities are inhibited. HsfA4 controls the ROS levels and is considered as an antiapoptotic factor, while HsfA5 specifically represses HsfA4 by inhibiting its DNA binding activity and acting as a proapoptotic factor^16^. HsfA3 is involved in drought and salt stress responses and is located in downstream of the DREB2A transcriptional cascade system^17^. In comparison to class A Hsfs, class B Hsfs have no typical transcriptional activity as they lack of an activator domain^2^. In tomato, the HS-induced HsfB1 act as a coactivator of HsfA1a by assembling into an enhanceosome-like complex^14^. Tomato HsfA1a, HsfA2, and HsfB1 form a triad to activate the expression of HS-inducible genes during HS response and recovery^2^. But HsfB1 from *Arabidopsis* is described as a repressor of HS-responsive Hsfs^18^. This indicates the Hsf members, even the orthologous genes, play divergent roles in different species. Recently, genome-wide expression profiles analyses of *Hsfs* were performed in many plant species, including two woody species *P. trichocarpa* and *S. suchowensis*^13^,^19^. However, little is known about *Hsfs* in the desert poplar, *P. euphratica*.

*P. euphratica*, along with the two other genome sequenced woody species, *P. trichocarpa* and *S. suchowensis*, belong to the Salicaceae family. Their morphological characteristics and stress tolerance performances were significantly different. As a native species in desert regions, *P. euphratica* shows distinguished adaptability to various abiotic stresses^20^.

To explore the potential roles of *P. euphratica Hsfs* in abiotic stress responses, the current study identified 32 *PeuHsf* genes and analyzed their evolutionary relationships, gene structures, conserved domains, *cis*-acting elements, and expression patterns across different tissues and under various abiotic stresses. In addition, the expression profiles of *PeuHsfs* under different abiotic stresses were compared to the well-studied *Hsfs* in *Arabidopsis*. In conclusion, the present study is helpful in understanding the distinguished stress tolerance of *P. euphratica* and providing the basis for further functional studies of *PeuHsfs*.

## Results

### Genome-wide identification of the *Hsf* genes in *P. euphratica*

To identify the *Hsf* genes in *P. euphratica*, the conserved Hsf domain (PF00447) from the Pfam database was used to search against the *P. euphratica* genome. In addition, the amino acid sequences of 27 AtHsfs and 31 PtHsfs were used as query to perform BLASTP search in the *P. euphratica* genome. After detection of the conserved DBD domain and the coiled-coil structure from the SMART database, a total of 32 *PeuHsfs* were identified (Table 1). The subfamily classification of PeuHsf was based on the length of the linker between DBD and HR-A/B regions and the amino acid length inserted into the HR-A/B regions. Moreover, the results were also confirmed in the Heatster database (http://www.cibiv.at/services/hsf/)^2^. The identified *PeuHsfs* encode proteins ranging from 207 to 737 amino acids (aa) in length (average of 384 aa), with molecular weight (MW) of 23.89 to 81.18 kDa (average of 42.91 kDa) and isoelectric point (pI) of 4.72 to 9.28. Among the 32 PeuHsf proteins, the percentage of negatively charged residues (Asp + Glu) ranged from 8.8% to 17.7% and the percentage of positively charged residues (Arg + Lys) ranged from 7.8% to 16.7%. Based on the instability index analysis, all the PeuHsf proteins were unstable. In addition, the aliphatic index had a range of 57.58 to 79.06 and the grand average of hydropathicity ranged from −0.903 to −0.415 (Table 1).

### Phylogenetic relationship of *PeuHsf* genes

**Figure 1:**
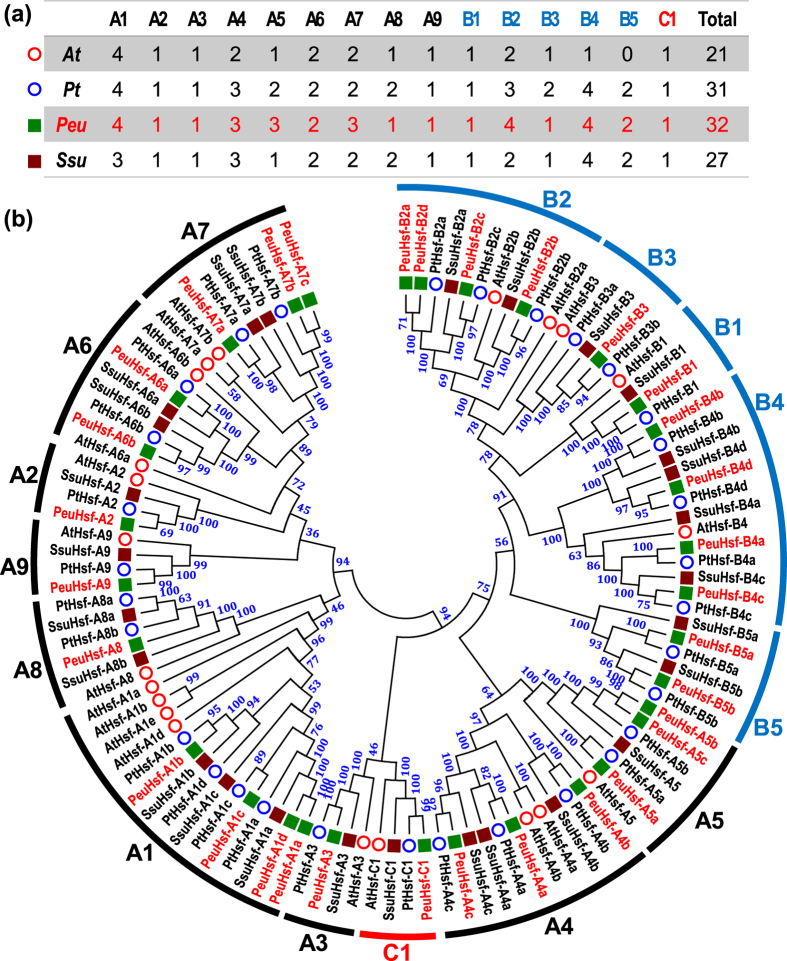
Hsf gene family and their phylogenetic relationships in *P. euphratica* (Peu), *P. trichocarpa (Pt*), *S. suchowensis (Ssu*), and *A. thaliana (At*). **(a)** The members of Hsf from four species were classified into three mainly subfamilies (class A, B, and C). **(b)** The phylogenetic tree was constructed using full-length of amino acid sequences of Hsf proteins in the four species by the neighbor-joining (NJ) method with 1,000 bootstrap replicates. Bootstrap support values are indicated on each node.

To explore the evolutionary characteristics of the *PeuHsf* genes, an unrooted phylogenetic tree was generated using the Hsf protein sequences from *P. euphratica, P. trichocarpa, S. suchowensis*, and *A. thaliana*. Compared to the two species in Salicaceae (*P. trichocarpa* and *S. suchowensis*) and model plant *Arabidopsis*, the desert poplar *P. euphratica* is the largest in size of the *Hsf* family (Fig. 1a). According to the phylogenetic tree, the *PeuHsfs* could be grouped into three main classes (A, B, and C) (Fig. 1b). Among the three main classes, class A was the largest class consisting of 19 members from nine subclasses (A1–A9), class B consisted of 12 members from five subclasses (B1–B5), while class C only contained one gene (Fig. 1). The length of class A PeuHsfs ranged from 331 to 737 aa (average of 449 aa), which was relatively longer than class B (207 to 368 aa with average of 285 aa) and class C (338 aa) (Table 1). All the three analyzed Salicaceae species contained *Hsf* members in subclass B5, while *Arabidopsis* did not include any member in subclass B5. Compared to the *Hsfs* in *P. trichocarpa, P. euphratica* containing one more in subclasses A5, A7, and B2, but one less in subclasses A8 and B3 (Fig. 1 and Supplementary Table S1).

### Structural analysis of PeuHsfs

#### Gene structures

**Figure 2:**
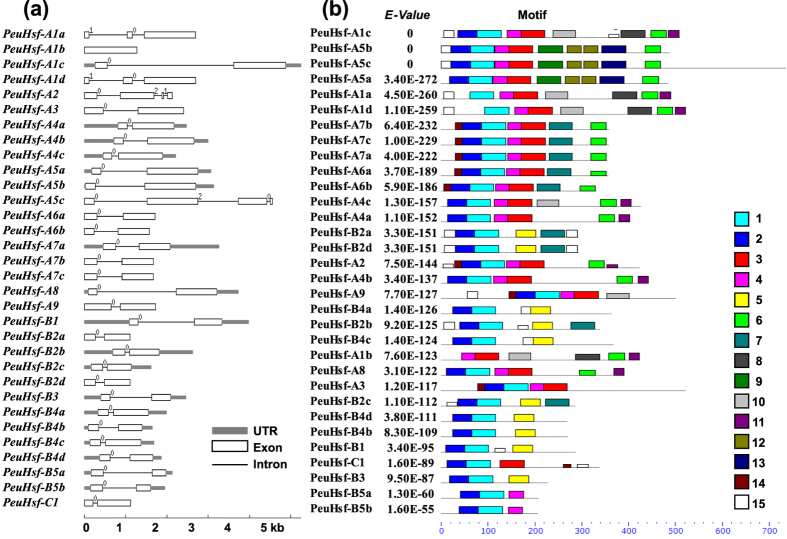
Gene structure (**a**) and conserved protein motifs (**b**) of members in *PeuHsf* family. **(a)** Grey box, blank box, and black line were represented UTR, exon, and intron, respectively. The number 0, 1, and 2 on the black line were intron phase. **(b)** Total of 15 conserved motifs were identified using MEME. The detail were listed in Supplementary Table S2.

The gene structures of *PeuHsfs* were analyzed by comparing the cDNA sequence and genomic DNA sequence. As shown in Fig. 2a, two *PeuHsfs (-A2* and *-A5c*) were comprised of four exons, two *PeuHsfs (-A1a* and *-A1d*) were comprised of three exons, while *PeuHsf-A1b* was only comprised of one exon without splice. Except for the aforementioned five *PeuHsfs*, all the other 27 *PeuHsfs* were comprised of two exons. Introns can be classified into phase 0, phase 1, and phase 2 depending on their position relative to the reading frame. All the two-exon containing *PeuHsfs* showed the same intron phase (phase 0). Over all, the gene structure and the intron phases were significantly conserved among the members in each subclass of *PeuHsf* family.

#### Conserved motifs

Based on the known information of Hsfs in *Arabidopsis*, six conserved domains including DBD, HR-A/B, NLS, NES, AHA, and RD were identified in PeuHsf proteins (Table 2). As the binding domain to the downstream functional genes, the DBD existed in all the PeuHsfs. The DBD was comprised of three α-helices and four β-sheets in the form of α1-β1-β2-α2-α3-β3-β4 (Supplementary Fig. S1). All the members of PeuHsf included NLS, which consists of nucleus localization of the transcription factor. With the exception of the members in subclasses A3 and A9, all the other class A PeuHsfs had NES domains. AHA motifs were only detected in class A PeuHsfs (except for PeuHsf-A9), four proteins in subclasses A2 and A4 had two AHA motifs, while PeuHsf-A3 had a longer AHA motif (W-X_17_-W-X_20_-W-X_15_-W) than the other members (Table 2).

In addition, the conserved motifs of PeuHsfs were also analyzed using MEME. Among the 15 detected motifs, motifs 1 and 2 existed in almost all of the PeuHsfs; the two motifs including the highly conserved DBD. Motif 3 represented coiled-coil structure in classes A and C PeuHsfs, while which was replaced by motif 5 in class B PeuHsfs. Moreover, motif 6 corresponding to the AHA motif nearby the C-terminus of class A PeuHsfs (Fig. 2b).

### Expansion of *Hsf* family in *P. euphratica*

#### Duplication events

To analyze the expansion of *Hsf* genes in *P. euphratica*, the *PeuHsfs* were mapped onto each scaffold based on publicly available information provided in the *P. euphratica* genome database. A total of 32 *PeuHsfs* were mapped onto 31 scaffolds (Supplementary Fig. S2). The synteny analysis was performed to identify the duplicated blocks. Based on the chromosomal location, duplicated blocks, phylogenetic relationships, gene structures, and conserved motifs of the *PeuHsfs*, total of 10 paralogous pairs were identified in *PeuHsf* family (Table 3). With the exception of one paralogous pair (*PeuHsf-A5b*/*PeuHsf-A5c*) that was generated by tandem duplication event, the other nine pairs were generated by whole genome duplication (WGD) (Supplementary Fig. S2). Two pairs (*PeuHsf-A1a*/*PeuHsf-A1d* and *PeuHsf-A5b*/*PeuHsf-A5c*) were generated in the latest stage (~0.49 MYA), three pairs (*PeuHsf-A4a*/*PeuHsf-A4c, PeuHsf-A6a*/*PeuHsf-A6b*, and *PeuHsf-A7a*/*PeuHsf-A7b*) were generated in ~13 MYA, and the other five pairs were generated between 17–20 MYA (Table 3). Comparatively, the generation date of *P. trichocarpa Hsf* paralogous pairs was relatively concentrated, one pair was generated in ~7.87 MYA, five pairs were generated in ~13 MYA, four pairs were generated in 17–20 MYA^19^.

Among the four analyzed species, 10, 10, 5, and 4 *Hsf* paralogous pairs were detected in *P. euphratica, P. trichocarpa, S. suchowensis*, and *A. thaliana*, respectively (Supplementary Table S3). With respect to the total number of *Hsfs* in each species, the proportions of duplicated *Hsfs* were relatively high in two *Populus* species (10 pairs in 31 *PtHsfs* or 32 *PeuHsfs*). Although the same number of *Hsf* paralogous pairs were identified in the allied species *P. euphratica* and *P. trichocarpa*, the composition of paralogous pairs was significantly different - two subclass A1 *Hsf* paralogous pairs in *P. euphratica* but only one pair in *P. trichocarpa, Hsf* paralogous pair in subclass B5 was specifically in *P. euphratica* while *Hsf* paralogous pairs in subclasses A8 and B3 were specifically in *P. trichocarpa* (Supplementary Table S3).

#### Ka/Ks values

**Figure 3:**
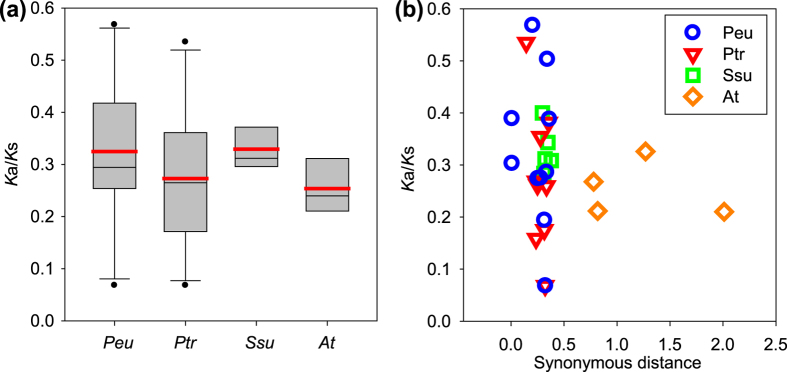
Estimates of *K*a/*K*s ratios in pairwise comparisons in four species *Hsf* families. **(a)** Average molecular evolutionary rate (*K*a/*K*s) for *Hsf* genes in *P. euphratica* (Peu), *P. trichocarpa* (Ptr), *S. suchowensis* (Ssu), and *A. thaliana* (At). Red line in each box indicates average value of *K*a/*K*s in each species. **(b)** The *K*a/*K*s ratios of the duplicated *Hsf* genes in four species are shown in the scatter plots, the y and x axes denote the *K*a/*K*s ratio and synonymous distance for each pair. The detail of the *K*a and *K*s in the four species was listed in Supplementary Table S3.

Subsequently, the substitution rate ratio of non-synonymous (*K*a) vs. synonymous (*K*s) substitutions was analyzed to verify the Darwinian position selection. The *K*a/*K*s ratios of all the 10 *PeuHsf* paralogous pairs were less than 0.6 (Table 3), indicating that the *PeuHsf* family had undergone purifying selection pressure. Compared to *S. suchowensis* and *A. thaliana*, the two *Populus* species had wide range of *K*a/*K*s, where the average *K*a/*K*s in *P. euphratica* was larger than that in *P. trichocarpa* (Fig. 3a). When combined, looking at the synonymous distance, we found that the synonymous distance of all the *Hsf* paralogous pairs were less than 0.5 in Salicaceae (*P. euphratica, P. trichocarpa*, and *S. suchowensis*) but more than 0.7 in *A. thaliana*, which resulted in the distribution of *AtHsf* paralogous pairs departing from *PeuHsf, PtHsf*, and *SsuHsf* paralogous pairs (Fig. 3b).

**Figure 4:**
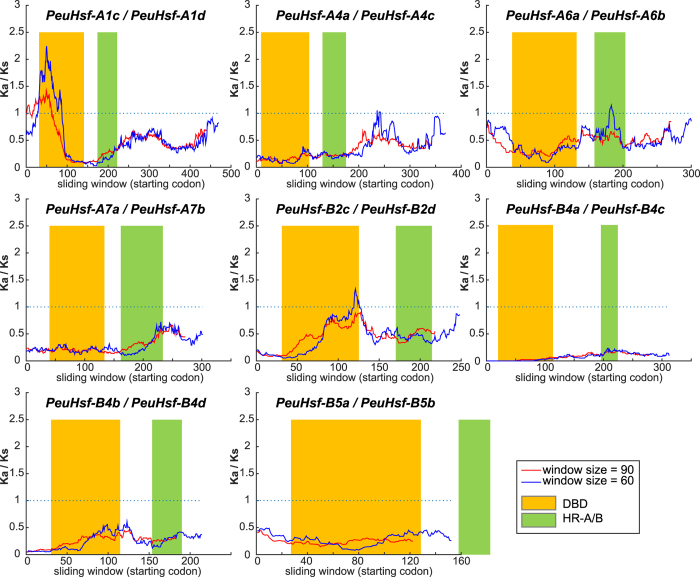
Sliding window plots of representative duplicated *Hsf* genes in *P. euphratica*. The window sizes were 90 bp and 60 bp separately.

Furthermore, a sliding window analysis of the *K*a/*K*s ratios was carried out for pairwise comparison. Two pairs (*PeuHsf-A1a*/*PeuHsf-A1d* and *PeuHsf-A5b*/*PeuHsf-A5c*) did not obtain the results because of their extremely similar sequences. Among the other eight *PeuHsf* paralogous pairs, the N-terminus of DBD in *PeuHsf-A1c*/*PeuHsf-A1d* pair had a significant peak higher than 1 that was under positive selection, while all the other regions were under purifying selection (Fig. 4). However, all the DBD and HR-A/B regions in *Arabidopsis Hsf* paralogous pairs were conserved with very low *K*a/*K*s ratio, except for the region behind HR-A/B in *AtHsf-A1a*/*AtHsf-A1c* pair had a peak more than 1 (Supplementary Fig. S3).

### Variety of *cis*-acting elements in the promoter regions of *PeuHsf* genes

**Figure 5:**
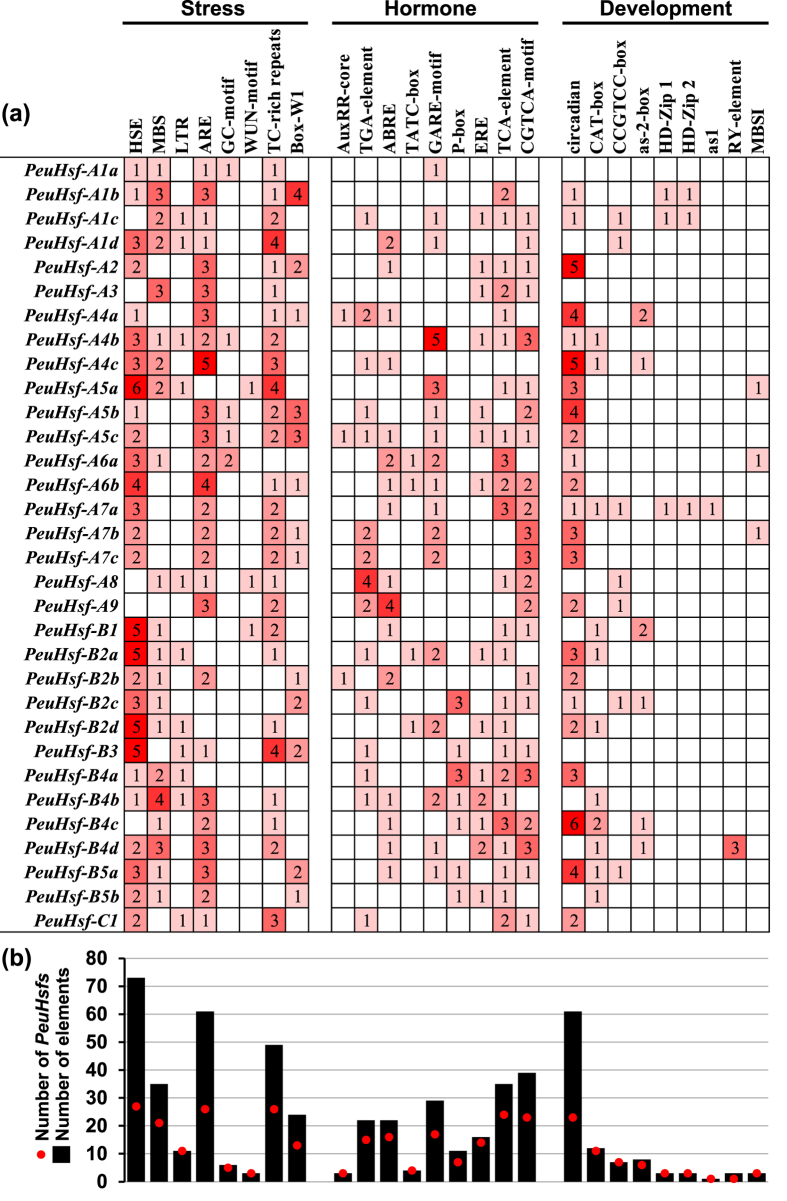
*cis*-acting elements in the promoter region of *PeuHsf* genes. **(a)** Number of each *cis*-acting element in the promoter region (2 kb upstream of translation initiation site) of *PeuHsf* genes. **(b)** The statistics of total number of *PeuHsf* genes including corresponding *cis*-acting elements (red dot) and total number of *cis*-acting elements in *PeuHsf* gene family (black box). Based on the functional annotation, the *cis*-acting elements were classified into three major classes: stress-, hormone-, or development-related *cis*-acting elements. (**HSE**, heat stress responsive element; **MBS**, MYB binding site involved in drought inducibility; **LTR**, low temperature responsive element; **ARE**, essential for the anaerobic induction; **GC-motif**, enhancer-like element involved in anoxic specific inducibility; **WUN-motif**, wound responsive element; **TC-rich repeats**, defense responsive element; **Box-W1**, fungal elicitor responsive element; **AuxRR-core** and **TGA-element**, auxin responsive element; **ABRE**, abscisic acid responsive element; **TATC-box**, **GARE-motif** and **P-box**, gibberellin responsive element; **ERE**, ethylene responsive element; **TCA-element**, salicylic acid responsive element; **CGTCA-motif**, MeJA responsive element; **circadian**, circadian control; **CAT-box**, meristem expression; **CCGTCC-box**, meristem specific activation; **as-2-box**, shoot-specific expression and light responsiveness; **HD-Zip1**, differentiation of the palisade mesophyll cells; **HD-Zip2**, control of leaf morphology development; **as1**, root-specific expression; **RY-element**, seed-specific regulation; **MBSI**, MYB binding site involved in flavonoid biosynthetic genes regulation.)

The 2 kb upstream sequences of the translation initiating site of the *PeuHsfs* were searched in the PlantCARE database to identify the potential *cis*-acting elements. As shown in Fig. 5, the identified *cis*-acting elements were classified into three mainly functional classes: stress, hormone, and development. In stress-related *cis*-acting elements, HSE, anaerobic induction element (ARE), TC-rich repeats, and MYB binding site involved in drought inducibility (MBS) were detected in the promoters of 27, 26, 26, and 21 *PeuHsfs*, respectively. Among these stress-related *cis*-acting elements, HSE was the most enriched element with enrichment level 2.7 (total of 73 HSE located in 27 *PeuHsfs* promoters, i.e. 73/27), followed by ARE (enrichment level 2.3), TC-rich repeats (enrichment level 1.9), and MBS (enrichment level 1.7). In hormone-related *cis*-acting elements, a total of 39 MeJA responsive elements (CGTCA-motif), 35 salicylic acid responsive elements (TCA-element), 29 gibberellin responsive elements (GARE-motif), 22 abscisic acid responsive elements (ABRE), and 22 auxin responsive elements (TGA-element) were detected in the promoters of 23, 24, 17, 16, and 15 *PeuHsfs*, respectively. Among the development-related *cis*-acting elements, 23 *PeuHsfs* had circadian related elements (enrichment level 2.7) and 16 *PeuHsfs* contained meristem related *cis*-acting elements (CAT-box or CCGTCC-box). Three *PeuHsfs* (-*A1b*, -*A1c*, and -*A7a*) had leaf development related elements (HD-Zip1 or HD-Zip2). In addition, three *PeuHsfs* (-*A5a*, -*A6a*, -*A7b*) contained MYB binding site involved in flavonoid biosynthetic gene regulation (MBSI).

### Expression profiles of *PeuHsfs* across different tissues and response to various abiotic stresses

#### Tissue-specific expression

**Figure 6:**
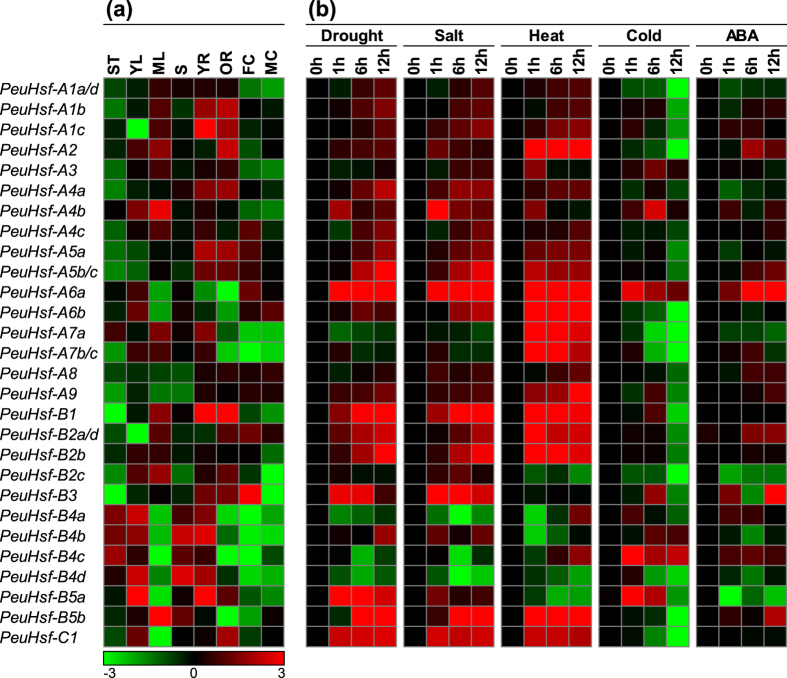
Expression patterns of *PeuHsf* genes across different tissues (**a**) and under various abiotic stresses (**b**) using qRT-PCR. **(a)** The expression of *PeuHsfs* in shoot tip (ST), young leaf (YL), mature leaf (ML), stem (S), young root (YR), old root (OR), female catkin (FC), and male catkin (MC) from *P. euphratica*. **(b)** The expression patterns of *PeuHsfs* after treated for 0, 1, 6, or 12 h under drought (25% PEG w/v), salt (300 mM NaCl), heat (42 °C), cold (4 °C), or 100 μM abscisic acid (ABA). To illustrate, the expression of *PeuHsfs* in different tissues were compared with the median value among these tissues. For different treatment, the expression of *PeuHsfs* in 1, 6, or 12 h were compared with the control in 0 h. The different colors correspond to log2 transformed value, green indicates down-regulation and red represents up-regulation. The expression patterns of orthologous *Hsf* genes in *Arabidopsis* across various tissues and under abiotic stresses were shown in Supplementary Fig. S4 and Supplementary Fig. S5. Primers used for qRT-PCR are listed in Supplementary Table S3.

We then analyzed the spatial and temporal expression profiles of *PeuHsfs* in eight tissues including shoot tip (ST), young leaf (YL), mature leaf (ML), stem (S), young root (YR), old root (OR), female catkin (FC), and male catkin (MC). Because of the highly similarity of four pairs (*A1a*/*A1d, A5b*/*A5c, A7b*/*A7c, B2a*/*B2d*), no gene-specific primers could be designed to distinguish their expression patterns in each of the pairs. Among the 28 analyzed *PeuHsfs*, three of four members in subclass B4 *PeuHsfs* were highly expressed in shoot tip. Eight *PeuHsfs* (-*A1b*, -*A1c*, -*A2*, -*A4b*, -*A7a*, -*B1*, -*B2c*, and -*B5b*) were highly expressed in mature leaf compared with those in young leaf. Four members in subclass B4 *PeuHsf* were highly expressed in stem. Eight *PeuHsfs* (-*A1b*, -*A1c*, -*A4a*, -*A5a*, -*A5b/c*, -*B1*, -*B3*, and -*B5a*) were highly expressed in both young root and old root. Noticeably, *PeuHsf-B3* was highly expressed in female catkin but scarcely expressed in male catkin (Fig. 6a).

#### Stress responses

To identify the roles of *PeuHsfs* in stress responses, the expression patterns of *PeuHsfs* were analyzed in the leaves of *P. euphratica* seedlings treated with drought (25% PEG w/v), salt (300 mM NaCl), heat (42 °C), cold (4 °C), or ABA (Fig. 6b). As the name declares, *PeuHsfs* were dramatically responsive to heat stresses, as a total of 10 *PeuHsfs* (-*A2*, -*A5b/c*, -*A6a*, -*A6b*, -*A7a*, -*A9*, -*B1*, -*B2b*, -*B5b*, and -*C1*) showed prompt induction by heat stress at 1 h. Aside from heat stress, most of the *PeuHsfs* (e.g. *PeuHsf-A4b, -A6a, -B1, -B3*, -*B5a*, -*B5b*, -*C1*) were responsive to both drought and salinity stresses. In contrast to the strong responsiveness of *PeuHsfs* to heat, drought or salt stresses, only three *PeuHsfs (-A6a, -B4c*, and *-B5a*) were promptly induced after 1 h under cold stress. It is important to note that *PeuHsf-A6a* was promptly and significantly induced by all the tested treatments in this study (Fig. 6b).

#### Expression divergence between paralogous pairs

**Figure 7:**
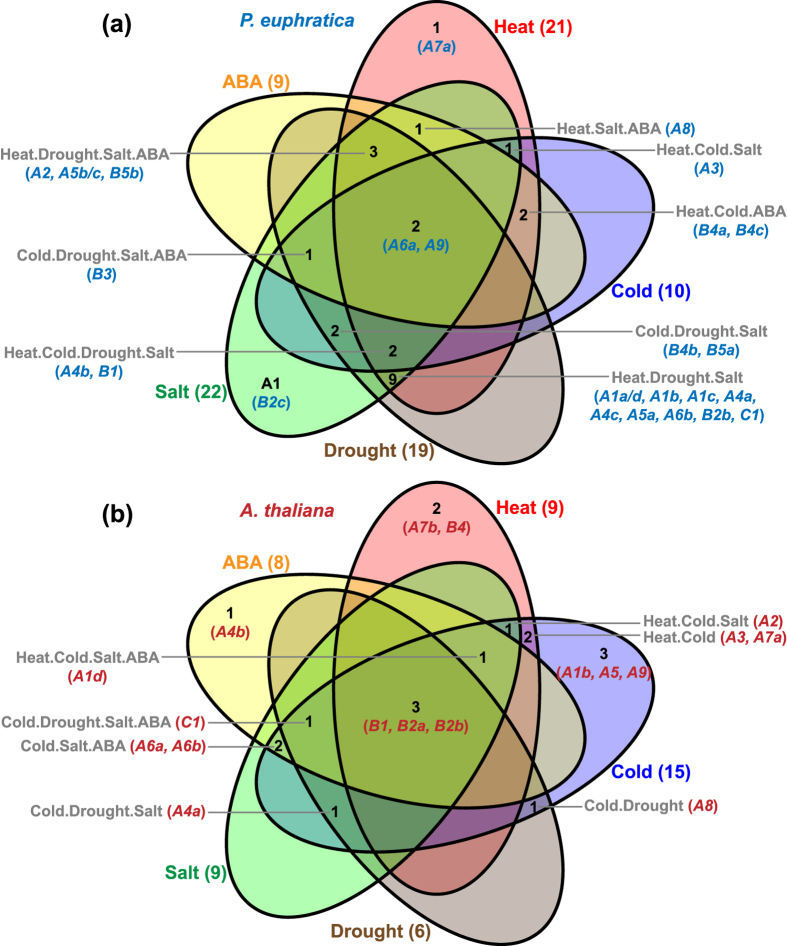
Different stress response model of *Hsf* family in *P. euphratica* (**a**) and *A. thaliana* (**b**). **(a)** The results were based on our qRT-PCR in Fig. 6b. **(b)** The results were based on the public available microarray data (see Supplementary Fig. S5).

The expression patterns of duplicated *PeuHsf* genes were divergent during the evolution. As indicated in Figs 6 and 7, most *PeuHsfs* in paralogous pairs showed different expression patterns. Among the 10 *PeuHsf* paralogous pairs, genes in two paralogous pairs (*PeuHsf-A1a*/*PeuHsf-A1d* and *PeuHsf-A5b*/*PeuHsf-A5c*) could not be effectively distinguished by qRT-PCR in our study because of their highly sequence similarity in each pair. The genes in three pairs (*PeuHsf-A6a*/*PeuHsf-A6b, PeuHsf-B4a*/*PeuHsf-B4c*, and *PeuHsf-B4b*/*PeuHsf-B4d*) showed similar expression patterns across different tissues (Fig. 6a), but had significant differences under various stresses (Fig. 6b). In contrast, the genes in the remaining five *PeuHsf* paralogous pairs presented different patterns both in tissues and under stress conditions (Fig. 6).

#### Divergence of expression patterns between P. euphratica and A. thaliana

To reveal the potential mechanism of distinguished stress tolerance of *P. euphratica*, the stress response profiles of *PeuHsfs* were also compared to *AtHsfs* (see Supplementary Fig. S5). As shown in Fig. 7, the responses of *Hsf* genes in the two species were significantly different. In *Arabidopsis*, with the exception of the 9 *Hsfs* that responded to heat stress, there were 15, 6, 9, and 8 *Hsfs* that were highly induced by cold, drought, salt, or ABA treatment, respectively (Fig. 7b). While in *P. euphratica*, more *Hsfs* were induced by these stresses. Total of 21 *PeuHsfs* were induced by heat stress. In addition, 22 and 19 *PeuHsfs* were induced by salt and drought stresses. Total of 10 and 9 *PeuHsfs* were induced by cold and ABA treatments. The orthologous genes in the two species showed significantly divergent expression patterns. For example, *AtHsfA2* was induced by heat, cold, and salt stresses but *PeuHsf-A2* was induced by heat, drought, salt, and ABA treatments. In *Arabidopsis*, three class B *AtHsfs* (-*B1*, -*B2a*, and -*B2b*) were widely induced by heat, cold, drought, salt, and ABA treatments; while in *P. euphratica*, only two class A *PeuHsfs* (-*A6a* and -*A9*) were induced by all the five treatments (Fig. 7).

## Discussion

As a class of ubiquitous transcription factors, the *Hsf* gene family plays an important role in various biological processes including plant development and stress tolerance. In this study, the evolution and divergence of the *Hsf* genes in desert poplar, *P. euphratica*, were studied to identify specific and subtle changes in these genes resulting in subfunctionalization or possible neofunctionalization.

After a comprehensive analysis, a total of 32 *Hsf* genes were identified in *P. euphratica* (Table 1). The size of the *Hsf* family in *P. euphratica* was larger than that of other species in Salicaceae such as *P. trichocarpa* and *S. suchowensis*^13^,^19^. Phylogenetic analysis revealed that *Hsf* genes in *P. euphratica* followed a similar distribution pattern as in other plant species (Fig. 1). Based on the previous classification, the *PeuHsfs* was classified as three main classes (A, B, and C). Classes A and B were further divided into A1-A9 and B1-B5 subclasses. Differing from *P. trichocarpa, P. euphratica* is a native species in desert area and has great tolerance to drought and salinity^20^. Compared with the stress sensitive poplar *P. trichocarpa*, the size of *Hsf* subclasses A5, A7, and B2 was larger in *P. euphratica* (Fig. 1). Meanwhile, the members in these subclasses had abundant stress-related *cis*-acting elements (Fig. 5) and showed significant stress responses (Fig. 6), which might contribute to the distinguished stress tolerance in *P. euphratica*.

It has been reported that the intron densities were significantly lower in the genes with rapid expression induction in response to stresses. Introns affect the expression efficiency through at least three possible aspects: delay transcript production by 1) splicing, 2) the added length of nascent transcript, or 3) the added energetic cost from increased transcript length^21^. Our analysis suggests that most of *PeuHsfs* contain one or two introns, which is helpful to their prompt induction under stress conditions. Interestingly, *HsfA2* in *P. euphratica* has three introns, while its orthologous genes have two and one intron(s) in *P. trichocarpa* and *S. suchowensis*, respectively^13^,^19^.

Gene duplication plays a critical role in the generation of new genes, increasing the number of these genes, and dispersing them in the genome^22^. In this study, the expansion of *PeuHsf* family was primarily due to the WGD events. The duplication events in *PeuHsf* gene family might introduce neofunctionalization, subfunctionalization, or nonfunctionalization. Most duplicated *PeuHsfs* display different spatial expression patterns or stress responses (Fig. 6), indicating that *PeuHsfs* are going through subfunctionalization or neofunctionalization. As dioecious and cross-pollinated plants, the gene flows in Salicaceae plants were stronger than self-pollinated plants such as *Arabidopsis*, which provided chance for subfunctionalization or neofunctionalization of duplicated genes. Despite *P. euphratica, P. trichocarpa*, and *S. suchowensis* belonging to the Salicaceae family, the genome sizes of *P. euphratica* and *P. trichocarpa* are significantly larger than *S. suchowensis*, which might be the reason for the broad range of *K*a/*K*s value in *P. euphratica* and *P. trichocarpa* (Fig. 3).

During the long stage of evolution, selection pressure plays an important role in shaping gene families, resulting in different evolutionary patterns among gene families or even in one gene family^23^. Our study demonstrates that the *K*a/*K*s ratios in *PeuHsf* HR-A/B regions were smaller than those in DBD domains (especially in paralogous pairs *PeuHsf-A1c/PeuHsf-A1d* and *PeuHsf-B2c/PeuHsf-B2d*) (Fig. 4), indicating that the HR-A/B region is relatively conserved in *PeuHsf* family. As the recognition and binding region, DBD domains play crucial roles in determining the specific downstream HS-inducible genes. The relatively high variation in DBD domains between *PeuHsf* paralogous pairs is helpful in enhancing the binding specificity and diversity of the *P. euphratica Hsf* family.

Among various *cis*-acting elements in promoter regions of *PeuHsfs*, HSE was the most abundant *cis*-acting element (Fig. 5). In the 32 members of *PeuHsf* family, total of 27 *PeuHsf* including 73 HSE in their promoter regions, and with high enrichment level (2.7). Based on our expression analysis (Fig. 6), 12 *PeuHsfs (-A2, -A6a, -A6b, -A7a, -A7b, -A7c, -B1, -B2a, -B2b, -B2d, -B5b*, and *-C1*) were highly induced by heat stress, which including 37 HSE in their promoter regions with a relatively higher enrichment level (3.1) than that in all HSE-including genes (2.7). These results suggest that there is a positive correlation between the *cis*-acting elements and gene expression patterns in *PeuHsf* gene family.

In *P. euphratica*, more than 60% *Hsf* genes were induced by heat, drought, or salt stresses (21, 19, and 22 *PeuHsfs*, respectively), which were significantly more than that in *Arabidopsis* (9, 6, and 9 *AtHsf*s, respectively) (Fig. 7). The expression patterns of *PeuHsfs* might be the evolved adaptive mechanism of *P. euphratica* to face the frequent heat, drought, and salt stresses in desert areas. In tomato and *Arabidopsis, HsfA2* has a high activator potential to activate the expression of *Hsp* genes. It has been identified as the dominant *Hsf* and strongly accumulated under heat stress^7^. In *P. euphratica*, the *PeuHsfA2* was also highly induced by heat stress, reaching ~500 folds at 1 h after heat stress (Fig. 6). The result implied that *HsfA2* might also be the dominant *Hsf* in *P. euphratica* similar to the tomato and *Arabidopsis*.

The members in *Hsf* family showed different stress responses across various plant species. The *P. euphratica HsfA2* was responsive to heat, drought, salt, and ABA treatments (Figs 6 and 7), while*Arabidopsis HsfA2* was responsive to heat, cold, and salt stresses (Fig. 7, Supplementary Fig. S5), indicating that *HsfA2* might be involved in ABA-independent pathway in *Arabidopsis* but ABA-dependent pathway in *P. euphratica*. Noticeably, some heat-insensitive *Hsfs* were involved in other stress tolerances. For instance, overexpression of wheat *HsfA4a* enhanced Cd tolerance but did not improve thermotolerance in transgenic rice^24^. In *Arabidopsis, HsfA4a* did not response to heat, but could be induced by cold, drought, and salt stresses (Fig. 7b). Consistently, overexpression of a dominant negative mutant form of *AtHsfA4a* increased oxidative stress sensitivity in transgenic *Arabidopsis*^25^. In contrast, *HsfA4a* in *P. euphratica* was not only induced by drought and salt stresses, but also induced by heat stress. Moreover, the other members in A4 subclass (*PeuHsfA4b* and *PeuHsfA4c*) were also induced by both heat and other stresses (Fig. 7a). It implies that these *Hsf* genes showed wide stress response patterns in the desert poplar.

It has been revealed that HsfA1 and HsfA2 can form a superactivator complex to activate downstream genes in a stronger manner than individual factors^10^, although overexpression of *AtHsfA2* also improved thermotolerance in quadruple knock-out *Arabidopsis* mutant (*athsfA1a, b, d, e*)^15^. In *Arabidopsis*, only one member (*A1d*) in A1 subclass showed multiple stress response to heat, cold, salt, and ABA (Fig. 7b), it could be cooperated with HsfA2 in these stress conditions. In contrast, many more A1 members (*A1a/d, A1b*, and *A1c*) in *P. euphratica* showed multiple stress responses to heat, drought, and salt stresses, and is similar to the expression patterns of *PeuHsfA2* (response to heat, drought, salt, and ABA treatments) (Fig. 7a). The high similarity of expression patterns of *PeuHsfA1s* and *PeuHsfA2* can form more superactivator complexes under heat, drought, and salt stresses in *P. euphratica*, which might be one reason for high stress tolerances of *P. euphratica* in arid and saline environments.

In conclusion, identification and detailed analysis of the *Hsf* gene family has been carried out in desert poplar, *P. euphratica*. Analysis suggests that *Hsf* gene family in *P. euphratica* has diverged during evolution and is widely responsive to various abiotic stresses. These findings are helpful in understanding the distinguished adaptability of *P. euphratica* to severe desert environments and provide the basis for functional analysis of *PeuHsfs* in the future.

## Methods

### Identification of Hsfs in *P. euphratica* and other plant species

For Hsf identification, the Hsf domain (PF00447) from the Pfam database (http://pfam.xfam.org/) was used to search against the *P. euphratica* genome (http://me.lzu.edu.cn/stpd/#main_tabs=0)^20^. In addition, the Hsf protein sequences of *A. thaliana*^26^ and *P. trichocarpa*^19^ were used as queries to perform a BLASTP search against the *P. euphratica* genome. The Simple Modular Architecture Research Tool (SMART, http://smart.embl-heidelberg.de/) was used to analyze the DBD domain and the coiled-coil structure.

### Sequence analysis

Full-length protein sequences of Hsf from four species, including model species *A. thaliana* and three Salicaceae species - *P. euphratica* (this study), *P. trichocarpa*^19^, and *Salix suchowensis*^13^, were aligned using the Clustal X2.1^27^ and the phylogenetic tree was constructed using MEGA 5^28^ with the neighbor-joining (NJ) method with 1,000 bootstrap replicates. The *P. euphratica Hsfs (PeuHsfs*) were named according to their subfamily classification and their phylogenetic relationships with the AtHsfs and PtHsfs (see Supplementary Table S1). The gene structures including exon and intron were displayed using Gene Structure Display Server (GSDS, http://gsds.cbi.pku.edu.cn/index.php). The conserved motifs of PeuHsfs were defined by Multiple Em for Motif Elicitation (MEME, http://meme-suite.org/). The parameters (e.g. molecular weight, isoelectric point, instability index, aliphatic index, GRAVY, and so on) of PeuHsf proteins were obtained from the ExPASy database (https://www.expasy.org/).The instability index provides an estimate of the stability of the protein in a test tube. A protein whose instability index is smaller than 40 is predicted as stable, a value above 40 predicts that the protein may be unstable. The aliphatic index of a protein is described as the relative volume occupies by the amino acids such as alanine, valine, isoleucine and leucine, which have an aliphatic side chain in their structure. The GRAVY value for a protein or a peptide is calculated by adding the hydropathy value of each amino acid residues and dividing by the number of residues in the sequence or length of the sequence. Increasing positive score indicates a greater hydrophobicity.

### Chromosome location and duplication analysis

The *PeuHsf* genes were mapped onto *P. euphratica* scaffolds based on the publicly available information provided in the *P. euphratica* genome database. For duplication of *Hsf* genes in *A. thaliana* and *P. trichocarpa*, the duplication events were obtained from the Plant Genome Duplication Database (http://chibba.agtec.uga.edu/duplication/). For duplication of *Hsf* genes in *P. euphratica* and *S. suchowensis*, the duplicated events were identified based on the duplicated block from synteny analysis using MicroSyn^29^. A duplicated block was defined as a region where three or more conserved homologs were located within 15 genes up- and down-stream between scaffolds^30^.

### *In silico* analysis of *cis*-acting elements of *PeuHsfs*

The *cis*-acting elements in promoter region (2 kb upstream of translation initiation site) of the *PeuHsf* genes were identified using PlantCARE^31^.

### Calculation of *K*a/*K*s values

The paralogous pairs were aligned using Clustal X2.1 and analyzed using PAL2NAL (http://www.bork.embl.de/pal2nal/) to calculation the *K*a and *K*s substitution rates. The divergence time (T) was calculated according to T = *K*s/(2 × 9.1 × 10^−9^) MYA for *Populus*^22^.

### Plant growth conditions and treatments

*P. euphratica* were planted in the greenhouse at Chinese Academy of Forestry (Beijing, China). For different tissues, shoot tip (ST), young leaf (YL), mature leaf (ML), stem (S), young root (YR), old root (OR), female catkin (FC), and male catkin (MC) from *P. euphratica* were collected. For various abiotic stresses, 4-month-old *P. euphratica* seedlings were water-cultured using Hoagland solution^32^. The seedlings were treated with 25% (w/v) polyethylene glycol 6000 (PEG6000, for drought stress), 300 mM NaCl (for salt stress), 42 °C (for heat stress), 4 °C (for cold stress), or 100 μM abscisic acid (ABA). The dosages of the abiotic stresses and hormone treatment were determined based on treatments in poplar^19^,^33^. Considering the outstanding salt tolerance of *P. euphratica*, the concentration of NaCl was increased to 300 mM from 150 mM. The leaves mixed from eight individuals at four time points (0, 1, 6, and 12 h) during the treatments were collected and frozen immediately in liquid nitrogen until to use. Three biological replicates were performed for each sample.

The expression data of *Arabidopsis Hsf* genes response to various abiotic stresses and hormone was download from AtGenExpress database (http://jsp.weigelworld.org/expviz/expviz.jsp). The *Arabidopsis* seedlings were treated under 38 °C (for heat stress, and recovery at 25 °C), 4 °C (for cold stress), 150 mM NaCl (for salt stress), 300 mM mannitol (for osmotic stress), 1.5 μg/ml bleomycin + 22 μg/ml mitomycin (for genotoxic stress), 10 μM methyl viologen (for oxidative stress), punctured with pins (for wounding stress), or 10 μM ABA. For drought stress, the *Arabidopsis* seedlings were stressed by 15 min dry air stream (clean bench) until 10% loss of fresh weight, then incubation in closed vessels in the climate chamber^34^. To compare the responses of *Hsf* genes to various abiotic stresses and hormone treatment between *P. euphratica* and *A. thaliana*, the significantly induced genes were selected by the following criteria: *P* < 0.05 and the value of Log_2_ (fold change) ≥1.

### RNA extraction and qRT-PCR analysis

Total RNA was isolated from *P. euphratica* materials using the RNeasy Plant Mini Kit (Qiagen) according to the manufacture’s protocol. Approximately 4 μg of total RNA were reverse transcribed using the SuperScript III reverse transcription kit (Invitrogen) to generate cDNA. qRT-PCR was performed on the LightCycler^®^ 480 Real Time PCR System (Roche) using SYBR Premix Ex Taq^TM^ Kit (Takara) according to the manufacturer’s procedure. The *PeuActin* gene was used as an internal control. The final threshold cycle (Ct) values were the mean of four values for each sample and four technical replicates. All primers used in this study are listed in Supplementary Table S3.

### Statistical analyses

Statistical analyses of qRT-PCR were carried out using SPSS 16.0 software (SPSS Inc, Chicago, IL, USA). Data was compared using Student’s *t* test. Differences were considered to be significant if *P* < 0.05.

## Additional Information

**How to cite this article**: Zhang, J. *et al*. Molecular evolution and expression divergence of the *Populus euphratica Hsf* genes provide insight into the stress acclimation of desert poplar. *Sci. Rep.* **6**, 30050; doi: 10.1038/srep30050 (2016).

## Supplementary Material

Supplementary Information

## Figures and Tables

**Table 1:** The *Hsf* genes identified from the *P. euphratica*.

Gene Name	ID	Map position (bp)	Length (aa)	MW (kDa)	pI	n.c.r. (%)	p.c.r. (%)	I.I.	Stability	A.I.	GRAVY
*PeuHsf-A1a*	CCG006597.1	scaffold1541.1:1132–3828(+)	491	53.7	4.95	63 (12.8%)	41 (8.4%)	61.54	unstable	72.75	−0.553
*PeuHsf-A1b*	CCG033924.1	scaffold96.1:588160–589431(−)	423	46.6	5.53	50 (11.8%)	38 (9%)	65.03	unstable	65.93	−0.626
*PeuHsf-A1c*	CCG014424.1	scaffold25.1:1905477–1910713(−)	510	56	4.79	67 (13.1%)	45 (8.8%)	64.06	unstable	67.73	−0.647
*PeuHsf-A1d*	CCG024768.1	scaffold49.1:1143998–1146694(−)	523	57.2	4.95	64 (12.2%)	41 (7.8%)	62.77	unstable	76.33	−0.486
*PeuHsf-A2*	CCG009416.1	scaffold19.1:1223913–1226045(−)	425	48.1	5.04	65 (15.3%)	47 (11.1%)	53.2	unstable	71.29	−0.573
*PeuHsf-A3*	CCG023633.1	scaffold4512.1:713–3120(+)	522	58.2	4.78	82 (15.7%)	51 (9.8%)	66.9	unstable	65.17	−0.613
*PeuHsf-A4a*	CCG026694.1	scaffold56.1:184555–187033(+)	404	45.9	5.22	57 (14.1%)	43 (10.6%)	56.17	unstable	65.17	−0.801
*PeuHsf-A4b*	CCG015090.1	scaffold26.1:1129045–1132041(−)	444	51	5.62	64 (14.4%)	49 (11%)	62.36	unstable	65.43	−0.794
*PeuHsf-A4c*	CCG030319.1	scaffold739.1:61353–63562(−)	427	—	—	61 (14.3%)	45 (10.5%)	55.87	unstable	63	−0.772
*PeuHsf-A5a*	CCG001724.1	scaffold1039.1:58159–61226(−)	485	54.1	5.96	63 (13%)	56 (11.5%)	56.93	unstable	67.2	−0.783
*PeuHsf-A5b*	CCG019200.1	scaffold323.1:258192–261325(−)	489	54.6	5.92	65 (13.3%)	57 (11.7%)	59.16	unstable	70.43	−0.785
*PeuHsf-A5c*	CCG019201.1	scaffold323.1:281255–285809(−)	737	81.2	7.02	82 (11.1%)	81 (11%)	54.67	unstable	77.84	−0.577
*PeuHsf-A6a*	CCG007863.1	scaffold17.1:1250083–1251797(−)	358	41.4	5.14	58 (16.2%)	43 (12%)	54.42	unstable	67.23	−0.788
*PeuHsf-A6b*	CCG000940.1	scaffold1.1:8456623–8458194(+)	331	38.2	5.08	56 (15.8%)	41 (11.5%)	59.91	unstable	72.15	−0.736
*PeuHsf-A7a*	CCG013195.1	scaffold23.1:595273–598538(−)	355	40.2	5.33	55 (15.5%)	45 (12.7%)	66.56	unstable	64.31	−0.882
*PeuHsf-A7b*	CCG017804.1	scaffold30.1:760950–762628(−)	359	41.2	5.44	54 (15%)	47 (13.1%)	55.04	unstable	68.75	−0.788
*PeuHsf-A7c*	CCG034159.1	scaffold988.1:37988–39666(−)	359	41.2	5.44	54 (15%)	47 (13.1%)	55.25	unstable	68.75	−0.782
*PeuHsf-A8*	CCG033049.1	scaffold9.1:2064027–2067752(−)	392	44.7	4.72	66 (16.8%)	37 (9.4%)	43.46	unstable	72.6	−0.698
*PeuHsf-A9*	CCG013896.1	scaffold240.1:34097–35820(+)	501	55.8	5.28	76 (15.2%)	58 (11.6%)	57.98	unstable	71.86	−0.586
*PeuHsf-B1*	CCG009216.1	scaffold188.1:576141–580111(+)	288	31.4	4.73	51 (17.7%)	35 (12.2%)	38.99	unstable	58.26	−0.903
*PeuHsf-B2a*	CCG001341.1	scaffold1000.1:34084–35186(+)	294	32.4	5.02	44 (15%)	34 (11.6%)	49.29	unstable	68.54	−0.669
*PeuHsf-B2b*	CCG024466.1	scaffold48.1:934135–936766(−)	340	36.6	4.95	47 (13.8%)	35 (10.3%)	53.42	unstable	69.76	−0.552
*PeuHsf-B2c*	CCG005948.1	scaffold148.1:57661–59281(+)	287	31.9	5.02	47 (16.4%)	37 (12.9%)	52.39	unstable	79.06	−0.507
*PeuHsf-B2d*	CCG007214.1	scaffold16.1:1230405–1231507(+)	294	32.4	5.02	44 (15%)	34 (11.6%)	49.29	unstable	68.54	−0.669
*PeuHsf-B3*	CCG001458.1	scaffold101.1:1374018–1376473(−)	228	26.5	7.62	37 (16.2%)	38 (16.7%)	55.59	unstable	72.28	−0.736
*PeuHs -B4a*	CCG027759.1	scaffold6.1:2805693–2807677(+)	364	40.4	8.15	32 (8.8%)	34 (9.3%)	51.71	unstable	73.9	−0.466
*PeuHsf-B4b*	CCG020996.1	scaffold38.1:136369–138015(+)	271	31.5	6.49	34 (12.5%)	31 (11.4%)	56.94	unstable	71.51	−0.585
*PeuHsf-B4c*	CCG010253.1	scaffold2.1:1006270–1007951(+)	368	41	7.72	33 (9%)	34 (9.2%)	55.68	unstable	69.16	−0.552
*PeuHsf-B4d*	CCG023235.1	scaffold44.1:1597707–1599573(−)	270	31.3	7.18	32 (11.9%)	32 (11.9%)	58.82	unstable	68.93	−0.651
*PeuHsf-B5a*	CCG006244.1	scaffold15.1:1276216–1278351(+)	209	24.1	9.24	26 (12.4%)	33 (15.8%)	55.35	unstable	71	−0.649
*PeuHsf-B5b*	CCG032077.1	scaffold84.1:65593–67546(−)	207	23.9	9.28	22 (10.6%)	30 (14.5%)	52.67	unstable	57.58	−0.744
*PeuHsf-C1*	CCG004861.1	scaffold135.1:519792–520919(+)	338	37.8	5.92	40 (11.8%)	37 (10.9%)	49.6	unstable	75.86	−0.415

Notes: pI, isoelectric point; n.c.r., total number of negatively charged residues (Asp + Glu); p.c.r., total number of positively charged residues (Arg + Lys); I.I., instability index; A.I., aliphatic index; GRAVY, grand average of hydropathicity.

**Table 2:** Functional domains of PeuHsfs.

Gene Name	DBD	HR-A/B	NLS	NES	AHA	RD
PeuHsf-A1a	33–110	143–190	(223) NKKRRLKQ	(475) VDQLTEQME	(432) SSFWDDLLVQ	N.D.
PeuHsf-A1b	1–35	61–108	(141) SKKRRLPR	(409) MNRLAEQMG	(361) DVFWEQFLTA	N.D.
PeuHsf-A1c	33–126	159–206	(239) NKKRRLKQ	(493) MDQLTEQMG	(450) SSFWDDLLAQ	N.D.
PeuHsf-A1d	78–142	175–222	(255) NKKRRLKQ	(507) VDQLTEQME	(464) SSFWDDLLVQ	N.D.
PeuHsf-A2	40–133	157–201	(229) RR-X_8_-RKRR	(411) LDSTALYVGFL	(318) ETIWEEFLTD; (357) DWSDDFQE	N.D.
PeuHsf-A3	90–183	209–253	(270) ARLKQKKEQ	N.D.	(446) W-X_17_-W-X_20_-W-X_15_-W	N.D.
PeuHsf-A4a	10–103	129–171	(204) DRKRRL	(391) LTEQMGHL	(257) LTFWENMVHD; (340) DVFWEQFLTE	N.D.
PeuHsf-A4b	11–104	125–178	(203) NKKRKL	(431) LAKNMGHI	(253) LKFLEDFLYA; (378) DLFWQHFLTE	N.D.
PeuHsf-A4c	10–103	129–174	(204) DRKRRL	(394) LTEQMGHL	(258) LTFWENMVND; (343) DVFWEQFLTE	N.D.
PeuHsf-A5a	14–107	126–177	(196) SK-X_10_-KKRR	(480) MEQLSL	(433) DGFWEQFLTE	N.D.
PeuHsf-A5b	18–111	133–181	(200) RK-X_10_-KKRR	(484) MEQLSL	(438) DVFWEQFLTE	N.D.
PeuHsf-A5c	18–111	133–181	(200) RK-X_10_-KKRR	(484) MEQLSL	(438) DVFWEQFLTE	N.D.
PeuHsf-A6a	40–133	159–197	(238) KKKRR	(346) LVEQLGYL	(322) EAFWEDLLNE	N.D.
PeuHsf-A6b	17–110	138–180	(215) KKRRR	(317) LGSEGED	(299) EGFWEDLLNE	N.D.
PeuHsf-A7a	42–135	163–234	(231) KKKELEEAMTKKRRR	(343) LAERMGYL	(322) EGFWEELLNE	N.D.
PeuHsf-A7b	42–135	163–208	(231) KRKELEEAMTKKRR	(344) LAERLGYL	(322) EGFWEELLNE	N.D.
PeuHsf-A7c	42–135	163–208	(231) KRKELEEAMTKKRR	(344) LAERLGYL	(322) EGFWEELLNE	N.D.
PeuHsf-A8	8–101	135–175	(161) NKLLLLRDR	(380) TEQMGLL	(298) DGSWEQLLLA	N.D.
PeuHsf-A9	157–250	272–313	(341) KR-X_12_-KKRR	N.D.	N.D.	N.D.
PeuHsf-B1	6–99	151–179	(255) LFGV-X_6_-KKKR	N.D.	N.D.	(255) LFGV
PeuHsf-B2a	27–120	161–189	(168) RLRK	N.D.	N.D.	(225) IFGV
PeuHsf-B2b	36–129	196–222	(282) LFGV-X_4_-KRVR	N.D.	N.D.	(282) LFGV
PeuHsf-B2c	32–125	173–214	(177) RLRK	N.D.	N.D.	(235) IFGV
PeuHsf-B2d	27–120	161–189	(168) RLRK	N.D.	N.D.	(225) IFGV
PeuHsf-B3	15–108	146–192	(195) LFGV-X_9_-RKRK	N.D.	N.D.	(195) LFGV
PeuHsf-B4a	21–114	190–218	(318) LFGV-X_4_-KKR	N.D.	N.D.	(318) LFGV
PeuHsf-B4b	21–114	156–188	(254) LFGV-X_4_-NKR	N.D.	N.D.	(254) LFGV
PeuHsf-B4c	21–114	198–221	(322) LFGV-X_4_-KKR	N.D.	N.D.	(322) LFGV
PeuHsf-B4d	21–114	152–186	(253) LFGV-X_4_-NKR	N.D.	N.D.	(253) LFGV
PeuHsf-B5a	29–131	164–182	(119) RGRR	N.D.	N.D.	N.D.
PeuHsf-B5b	28–128	158–182	(164) NKNLRR	N.D.	N.D.	N.D.
PeuHsf-C1	9–102	131–157	(193) KKQR	N.D.	N.D.	N.D.

N.D.: no motifs detectable by sequence similarity search.

**Table 3:** Divergence between paralogous *PeuHsf* gene pairs.

Subfamily	Gene 1	Gene 2	Type	*K*a	*K*s	*K*a/*K*s	Date (MYA)
*A1*	*PeuHsf-A1a*	*PeuHsf-A1d*	WGD	0.004	0.009	0.389	0.49
*A1*	*PeuHsf-A1c*	*PeuHsf-A1d*	WGD	0.173	0.343	0.503	18.85
*A4*	*PeuHsf-A4a*	*PeuHsf-A4c*	WGD	0.069	0.253	0.274	13.90
*A5*	*PeuHsf-A5b*	*PeuHsf-A5c*	Tandem	0.003	0.009	0.303	0.49
*A6*	*PeuHsf-A6a*	*PeuHsf-A6b*	WGD	0.116	0.204	0.568	11.20
*A7*	*PeuHsf-A7a*	*PeuHsf-A7b*	WGD	0.077	0.279	0.275	15.31
*B2*	*PeuHsf-B2c*	*PeuHsf-B2d*	WGD	0.141	0.362	0.388	19.91
*B4*	*PeuHsf-B4a*	*PeuHsf-B4c*	WGD	0.022	0.325	0.068	17.86
*B4*	*PeuHsf-B4b*	*PeuHsf-B4d*	WGD	0.061	0.316	0.194	17.38
*B5*	*PeuHsf-B5a*	*PeuHsf-B5b*	WGD	0.096	0.335	0.286	18.38
