# Supplementary material for: Molecular evolution and expression divergence of the Populus euphratica Hsf genes provide insight into the stress acclimation of desert poplar
Source: Sci Rep. 2016 Jul 18;6:30050. doi: 10.1038/srep30050 (PMC4948027; doi:10.1038/srep30050)
Supplement: Supplementary Information [file srep30050-s1.pdf]

Supplementary Information

Molecular evolution and expression divergence of the *Populus euphratica Hsf* genes provide insight into the stress acclimation of desert poplar

Jin Zhang[1,2,+,*], Huixia Jia[1,2,+], Jianbo Li[1], Yu Li[1], Mengzhu Lu[1,2], and Jianjun Hu[1,2,*]

[1] State Key Laboratory of Tree Genetics and Breeding, Key Laboratory of Tree Breeding and Cultivation of the State Forestry Administration, Research Institute of Forestry, Chinese Academy of Forestry, Beijing 100091, China

[2] Co-Innovation Center for Sustainable Forestry in Southern China, Nanjing Forestry University, Nanjing 210037, China

[*] Correspondence and requests for materials should be addressed to J.Z. (email: zhang007jin@163.com) and J.H. (email: hujj@caf.ac.cn).

[+] These authors contributed equally to this work.

Fig. S1

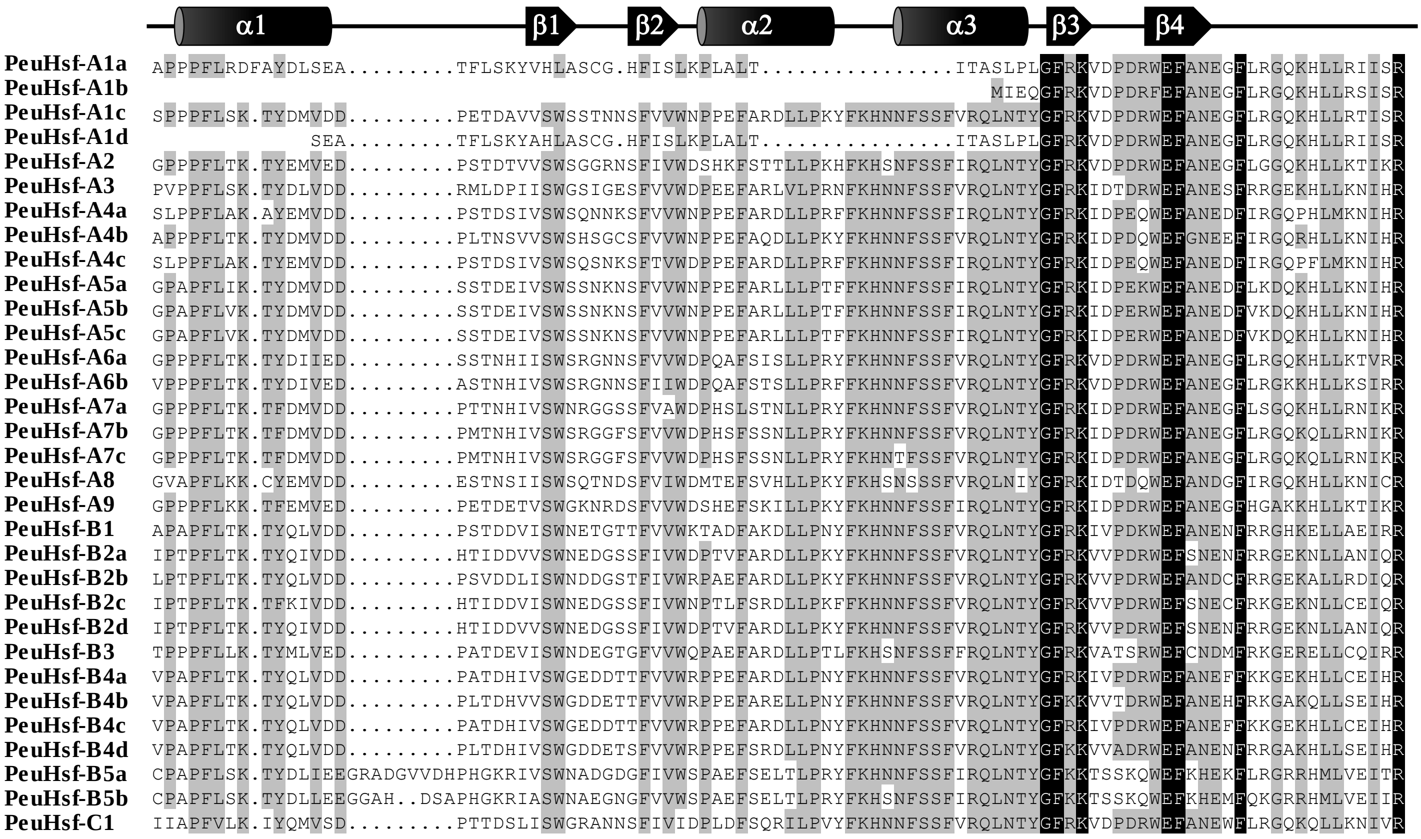

Fig. S1 Secondary structure of DBD region of *PeuHsf* family. Three α- helices and four β- sheets were presented in the region.

Fig. S2

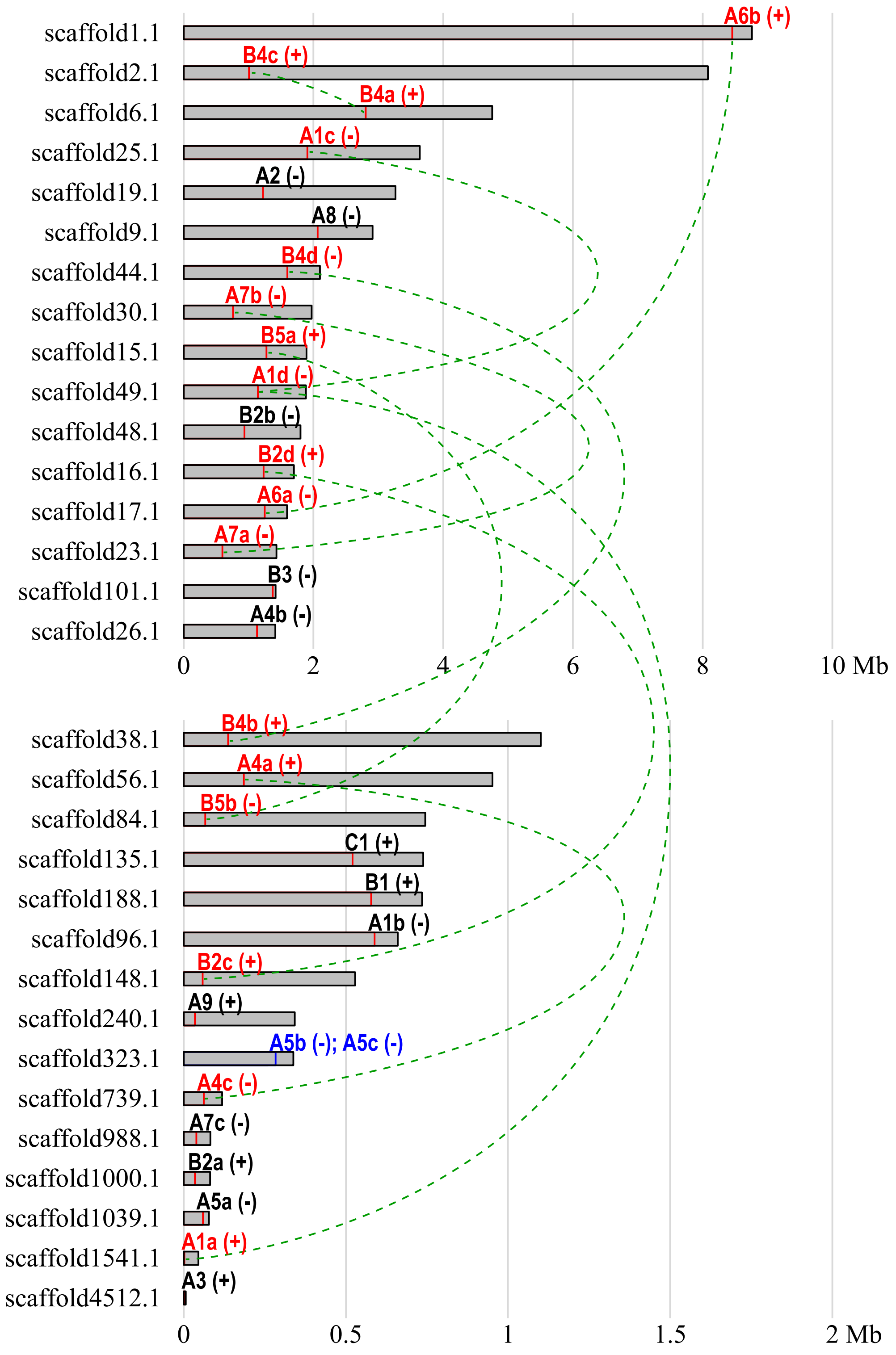

Fig. S2 Chromosomal locations of *PeuHsf* genes.

Total of 32 *PeuHsfs* are mapped onto 31 scaffolds. Red letters indicate duplicated genes generated by whole genome duplication (WGD) events, blue letters indicate tandem duplicated genes. Green dash lines connect duplicated *PeuHsfs* which generated by WGD events.

Fig. S3

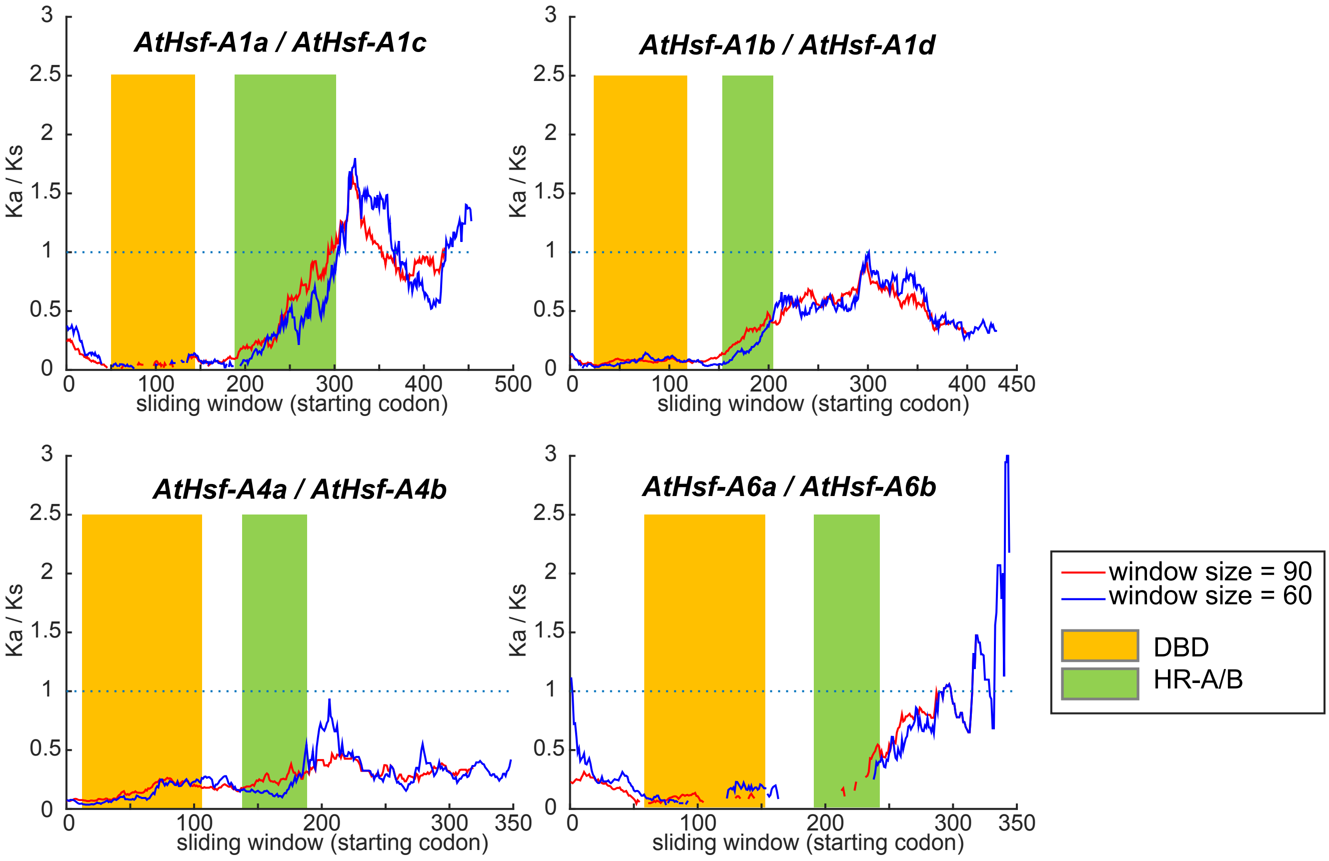

Fig. S3 Sliding window plots of representative duplicated *Hsf* genes in *A. thaliana*.

Fig. S4

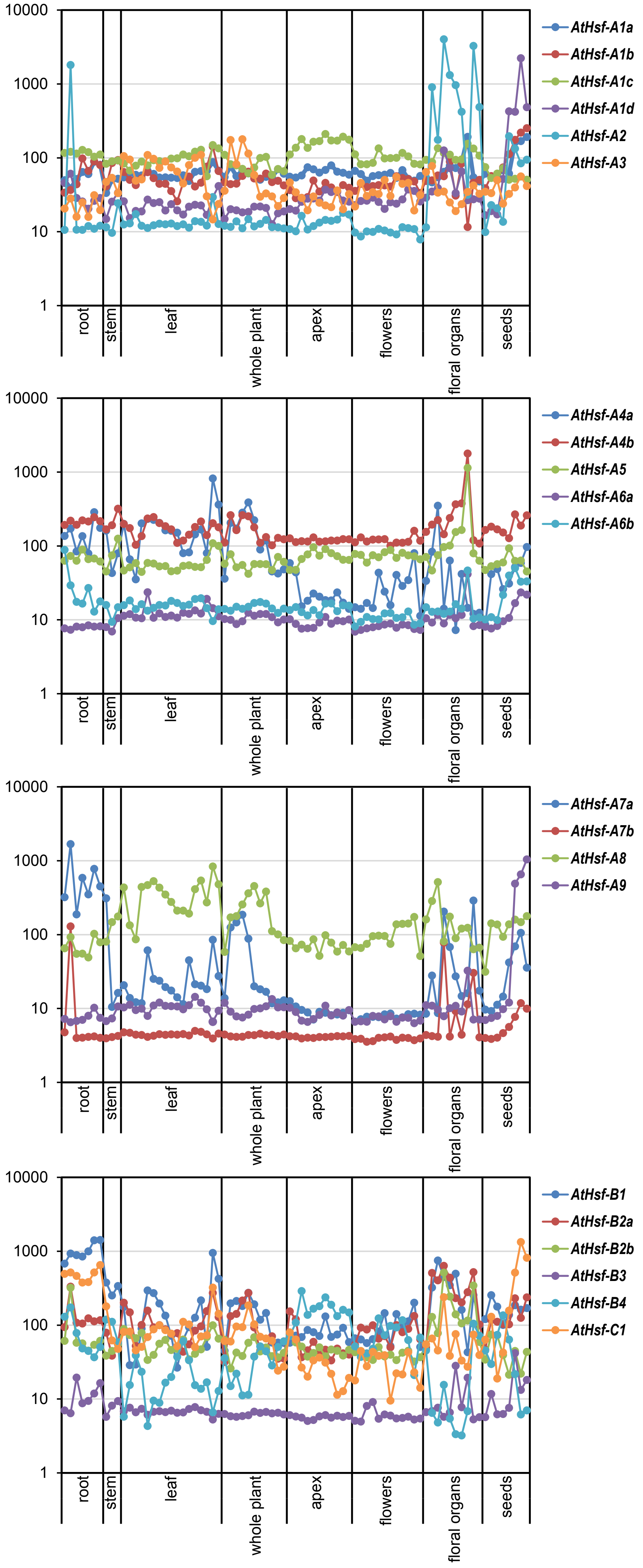

Fig. S4 Expression patterns of *Arabidopsis Hsf* genes across various tissues.

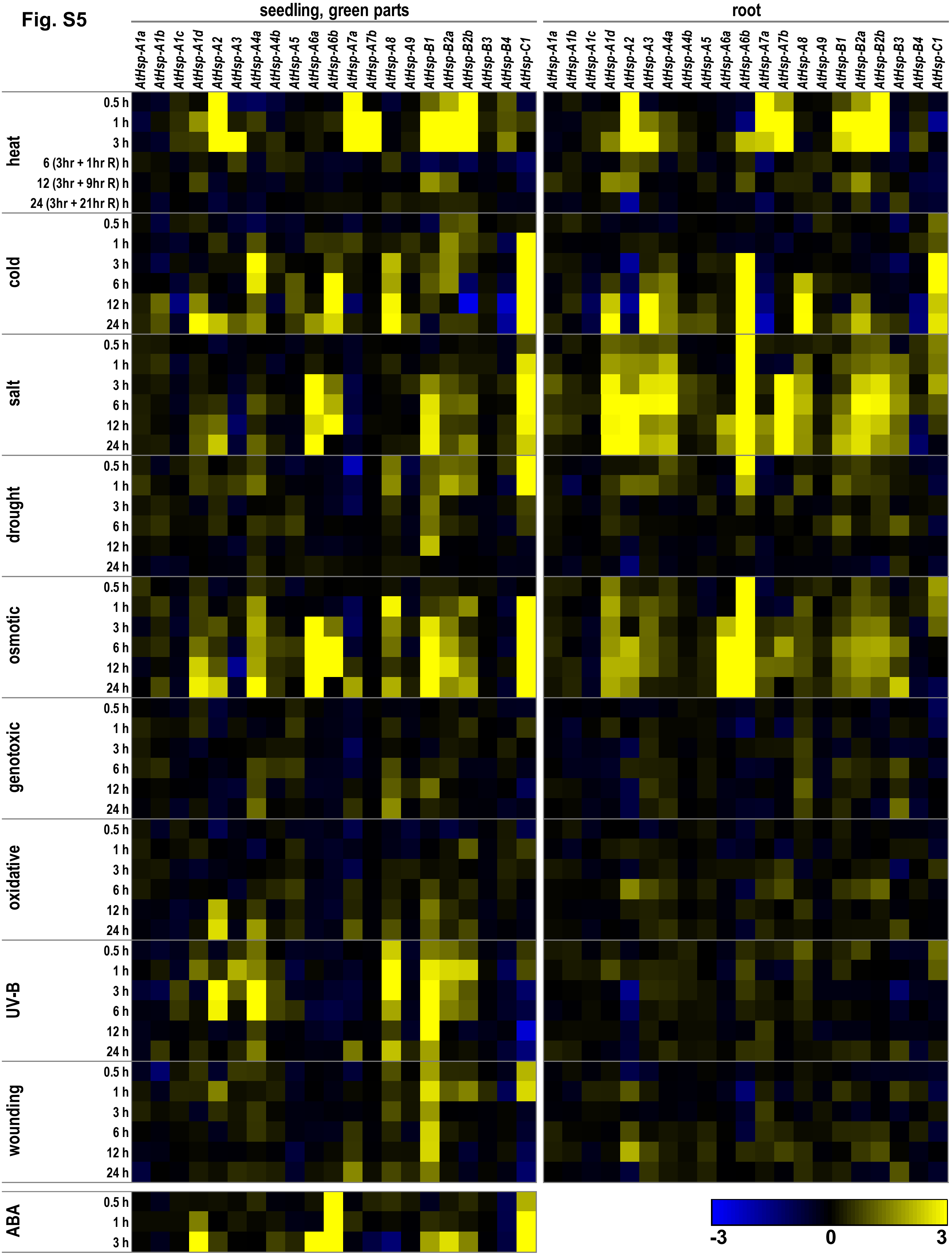

Fig. S5 Expression patterns of *Arabidopsis Hsf* genes across various abiotic stresses.

Table S1. Comparison of Hsf members in *P. euphratica*, *P. trichocarpa*, and *A. thaliana*.

	**Hsfs**		***P. euphratica* (Peu) 32**	***P. trichocarpa* (Pt) 31**	***A. thaliana* (At) 21**
Type A	A1	A1a	CCG006597.1	Potri.003G095000.1	At4g17750.1
		A1b	CCG033924.1	Potri.013G079800.1	At5g16820.1
		A1c	CCG014424.1	Potri.001G138900.1	At1g32330.1
		A1d	CCG024768.1	Potri.019G050400.1	At3g02990.1
	A2	A2	CCG009416.1	Potri.006G226800.1	At2g26150.1
	A3	A3	CCG023633.1	Potri.006G115700.1	At5g03720.1
	A4	A4a	CCG026694.1	Potri.011G071700.1	At4g18880.1
		A4b	CCG015090.1	Potri.014G141400.1	At5g45710.1
		A4c	CCG030319.1	Potri.004G062300.1	
	A5	A5a	CCG001724.1	Potri.017G059600.1	At4g13980.1
		A5b	CCG019200.1	Potri.001G320900.1	
		A5c	CCG019201.1		
	A6	A6a	CCG007863.1	Potri.010G082000.1	At5g43840.1
		A6b	CCG000940.1	Potri.008G157600.1	At3g22830.1
	A7	A7a	CCG013195.1	Potri.005G214800.1	At3g51910.1
		A7b	CCG017804.1	Potri.002G048200.1	At3g63350.1
		A7c	CCG034159.1		
	A8	A8a	CCG033049.1	Potri.008G136800.1	At1g67970.1
		A8b		Potri.010G104300.1	
	A9	A9	CCG013896.1	Potri.006G148200.1	At5g54070.1
Type B	B1	B1	CCG009216.1	Potri.007G043800.1	At4g36990.1
	B2	B2a	CCG001341.1	Potri.012G138900.1	At5g62020.1
		B2b	CCG024466.1	Potri.001G108100.1	At4g11660.1
		B2c	CCG005948.1	Potri.015G141100.1	
		B2d	CCG007214.1		
	B3	B3a	CCG001458.1	Potri.006G049200.1	At2g41690.1
		B3b		Potri.016G056500.1	
	B4	B4a	CCG027759.1	Potri.002G124800.1	At1g46264.1
		B4b	CCG020996.1	Potri.009G068000.1	
		B4c	CCG010253.1	Potri.014G027100.1	
		B4d	CCG023235.1	Potri.001G273700.1	
	B5	B5a	CCG006244.1	Potri.004G042600.1	
		B5b	CCG032077.1	Potri.011G051600.1	
Type C	C1	C1	CCG004861.1	Potri.T137400.1	At3g24520.1

Table S2. Motif sequences identified by MEME tools in PeuHsfs.

Motif	Length	Best possible match
1	50	RYFKHNNFSSFVRQLNTYGFRKIDPDRWEFANECFRRGQKHLLCNIHRRK
2	41	FLTKTYDMVDDPSTDHIVSWNRDGNSFVVWDPPEFARDLLP
3	50	VLMMELVRLRQQQQSTDHQIQAMEQRLQWMECRQQQMMSFLAKAMQNPGF
4	29	TQQQAVGACVEVGRFGYEEEIERLKRDKN
5	41	ALIEENERLRKENCMLMSELTHMKKLCNDIIYFVQNYVKPR
6	32	VNDVFWEQFLTESPGYGDIEEVSSCYKANGYD
7	49	MKKELEEAMTKKRRVPIDQKRGREGGGEMNHGEGHANPIKAEPREYGCY
8	50	IMPHVSQMPEMVPENVADIPCEDYMEPETCNDGFIDPASLGINGTIPIDI
9	50	AYSKKRRLPQVDHPMPIAENSLVENHCSSRPESNVIHQDFPDKLRLELSP
10	46	KKRRLKQEDVPENEGSGSHDGQIVKYQPLMNEAAKAMLRQIMKMDA
11	21	GWWKAQHVDNLTEQMGHLTPD
12	29	TQELNDTGGSYQFKINPGVPKDIPTRTSG
13	50	SNEEVDGHISCQLNLSLASSPLQVNKNPYLTRIPQLGQEIGKSPESRFNE
14	15	PPQPMEGLHDTGPPP
15	21	MQACTDHATCGGGGGQAAPWP

Numbers in the first column indicate the motifs represented in the Fig. 2b.

Table S3. Paralogous pairs in four species *Hsf* families.

	No.	Subfamily	Gene 1	Gene 2	Duplication	*K*a	*K*s	*K*a/*K*s
P. euphrarica	1	A1	*PeuHsf-A1c*	*PeuHsf-A1d*	W	0.173	0.343	0.503
	2	A1	*PeuHsf-A1a*	*PeuHsf-A1d*	W	0.004	0.009	0.389
	3	A4	*PeuHsf-A4a*	*PeuHsf-A4c*	W	0.069	0.253	0.274
	4	A5	*PeuHsf-A5b*	*PeuHsf-A5c*	T	0.003	0.009	0.303
	5	A6	*PeuHsf-A6a*	*PeuHsf-A6b*	W	0.116	0.204	0.568
	6	A7	*PeuHsf-A7a*	*PeuHsf-A7b*	W	0.077	0.279	0.275
	7	B2	*PeuHsf-B2c*	*PeuHsf-B2d*	W	0.141	0.362	0.388
	8	B4	*PeuHsf-B4a*	*PeuHsf-B4c*	W	0.022	0.325	0.068
	9	B4	*PeuHsf-B4b*	*PeuHsf-B4d*	W	0.061	0.316	0.194
	10	B5	*PeuHsf-B5a*	*PeuHsf-B5b*	W	0.096	0.335	0.286
P. trichocarpa	1	A1	*PtHsf-A1c*	*PtHsf-A1a*	W	0.098	0.277	0.354
	2	A4	*PtHsf-A4c*	*PtHsf-A4a*	W	0.063	0.236	0.268
	3	A5	*PtHsf-A5a*	*PtHsf-A5b*	O	0.063	0.235	0.269
	4	A6	*PtHsf-A6a*	*PtHsf-A6b*	W	0.077	0.143	0.535
	5	A7	*PtHsf-A7a*	*PtHsf-A7b*	W	0.065	0.249	0.261
	6	A8	*PtHsf-A8a*	*PtHsf-A8b*	W	0.087	0.334	0.260
	7	B2	*PtHsf-B2a*	*PtHsf-B2c*	W	0.135	0.355	0.382
	8	B3	*PtHsf-B3a*	*PtHsf-B3b*	W	0.037	0.235	0.158
	9	B4	*PtHsf-B4b*	*PtHsf-B4d*	W	0.055	0.314	0.176
	10	B4	*PtHsf-B4a*	*PtHsf-B4c*	W	0.022	0.321	0.068
S. suchowensis	1	A4	*SsuHsf-A4a*	*SsuHsf-A4c*	W	0.088	0.309	0.284
	2	A6	*SsuHsf-A6a*	*SsuHsf-A6b*	W	0.118	0.296	0.400
	3	A7	*SsuHsf-A7a*	*SsuHsf-A7b*	W	0.119	0.348	0.343
	4	A8	*SsuHsf-A8a*	*SsuHsf-A8b*	W	0.100	0.320	0.312
	5	B4	*SsuHsf-B4b*	*SsuHsf-B4d*	W	0.117	0.379	0.308
A. thaliana	1	A1	*AtHsf-A1a*	*AtHsf-A1c*	W	0.414	1.269	0.326
	2	A1	*AtHsf-A1d*	*AtHsf-A1b*	W	0.209	0.779	0.268
	3	A4	*AtHsf-A4a*	*AtHsf-A4b*	W	0.173	0.817	0.212
	4	A6	*AtHsf-A6a*	*AtHsf-A6b*	W	0.423	2.011	0.210

Table S4. List of primers used for qRT-PCR analysis.

	Forward primer	Reverse primer
PeuHsf-A1a/d	CAGCTTCTCATTTTCCGGATATG	GAAGCTGGATCTATGAATCCATCAT
PeuHsf-A1b	GGTTTCAGGAAGGTTGATCCTG	GTCTAACAAGTTCCTGCATGAGAAC
PeuHsf-A1c	GCCGCCATTTCTGAGTAAGACT	TAGCATGTCCATGGGCAGG
PeuHsf-A2	CCATCAAACGGAAAAGACATCTC	TTTCTCTCTGTGCTCCGTAACCTA
PeuHsf-A3	GTTGAAGAACATTCATAGGCGC	GCTGCCTGAAGTCTCTGATTTACT
PeuHsf-A4a	AGTTACAGCAAACGGAATGCAT	CATGGGAAGTCACCATTTGG
PeuHsf-A4b	ACCACATTCGAGGGACATTTG	CATTTGCTCCGTTCACCACA
PeuHsf-A4c	GCTGAAGCAAATGGAACGAAG	CAGAGCTTGGGAAGTAACCGTC
PeuHsf-A5a	GCATCTGCTTAAGAACATCCACA	GCTGTGAAAGCCCTAGAATACTTC
PeuHsf-A5b/c	CCACAGTCATAGTCAACCTCAAGGT	CCTCTGCTGCATAGTATCTATCTTCTG
PeuHsf-A6a	TAAGCCGAGACAAACGAGCAT	TCCTCTTCAAGCTCTTTCATCATG
PeuHsf-A6b	ACCAACTGATCAACTTGGTGGAG	AACACTCAACACATCTTCCTCATCTT
PeuHsf-A7a	CCTTTTTACAGCAGTTAGTTCAGCA	CCAGCTCTGAGACTTCAATTTCAAG
PeuHsf-A7b/c	GCAAAGGCTACAATGGACAGAA	ACCACCACTTCGACCAGGTCT
PeuHsf-A8	ACAGCTTTGTTATATGGGACATGAC	GCTCAATGGAGTTGTCTTGCTG
PeuHsf-A9	CAGCAGAGCGGAGCAGTTACT	GCAATTGTTTGCACTCTGCAG
PeuHsf-B1	GATCCAAGCACTGATGATGTGAT	GGAGCTCTTTGTGTCCTCGC
PeuHsf-B2a/d	CGATCATACGATAGACGATGTCG	GATTTTCCTCCTTTGTATATTGGCT
PeuHsf-B2b	CATGGAACGACGATGGATCA	AATATCGCGGAGAAGTGCTTT
PeuHsf-B2c	GGGAAACTCCAACATCCTCACTA	GGAGAAAAGGGTTGGATTCCAG
PeuHsf-B3	ATTTGTGGTGTGGCAACCG	GCAATAGGTTGTTGCTTGCTAGAC
PeuHsf-B4a	CTAATGGTTCTCTAGTACAGAAGCCC	CAATCTTTTCTTTGTTTGTAATGGC
PeuHsf-B4b	TGTCAGGCAGCTCAACACCTAC	CGCAGTTAGGATATGATCAACACA
PeuHsf-B4c	AGCACCATATGCTACTGCTAATCC	GCTCTGCGAAGTAGTTGTTGGA
PeuHsf-B4d	TTCAATCTCCAAAACCAAGTACTGA	GCTTAGGTGCTGCATTATAAGCC
PeuHsf-B5a	TTCAAGCATGAGAAGTTCTTGAGAG	TCTCCTCCATGAGTATCAAACGAT
PeuHsf-B5b	CATGATAGCGCTCCTCATGGT	CCTCCTCCCTTTCTGAAACATT
PeuHsf-C1	TCTCTCAGAGGATTTTGCCTGTT	ATGCTTCCTGCGAACTATATTCTTC
